# 

**Published:** 1891-08

**Authors:** 


### American Medical Editors' Association.

The Mississippi Valley Medical Association will meet in St. Louis, October 14, 15 and 16, 1891. Dr. C. H. Hughes, president, 500 N. Jefferson avenue, St. Louis, and Dr. E. S. McKee, secretary, 57 W. 7th street, Cincinnati, O., Dr. I. N. Love, chairman committee of arrangements, 301 N. Grand avenue, St. Louis.

The following letter from Dr. Hughes, who is also a member, and vice-president of the Association of American Medical Editors, and editor of the *Alienist and Neurologist*, has been issued to the medical press:

"ST. LOUIS, MO., August 3rd, 1891.

"*My Dear Doctor*:

"It has been proposed at the above date to have a meeting and conference of the Medical Press Association, and you are cordially invited to join us.

"Please give timely prominence to the date of meeting of the Mississippi Valley Medical Association, and favor us, if you can, with your presence on that occasion.

"A good and profitable time is expected, and we shall be glad to meet you. Yours truly,

"C. H. HUGHES, Vice-Prest. A. M. E. Ass'n.

"*To Dr. F. E. Daniel, Austin, Texas.*"

The Mississippi Valley Medical Association will hold its seventeenth annual session at the Pickwick theatre, Washington and Jefferson Aves., St. Louis, October 14, 15 and 16th. A full programme of interesting papers has been prepared and provision has been made for the fullest, freest and most complete discussion of the same. Representative men from various sections of the country have been invited to open the discussions. The local profession of St. Louis is a unit to the end that every visiting physician shall be received and welcomed in a regular warm hearted, St. Louis style.

The same qualifications are requisite for membership in this Association as for the American Medical Association, the former being subordinate to the latter. If eligible, you and your friends, together with your wives and families, are most cordially invited to visit St. Louis and enter into the scientific work and the social pleasures as you may desire.

I. N. LOVE, M. D.,
Chairman Committee Arrangements.

In connection with the above the JOURNAL is in receipt of a cordial invitation from Dr. E. S. McKee the Secretary, to all Texas physicians to attend the meeting. Hotel and railroad rates have been reduced and a royal good time is promised. Dr. McKee says: "All we need to make this Association a 'humming' success is a large sprinkling of whole-souled Texan Doctors with hearts as big as their hats, and ideas as broad as their boundless prairies. I hope, dear Doctor, that you will be with us, that you will bring plenty of doctors with, you and that you will keep before your readers the opportunity of their lives, the above mentioned meeting.

